# Bibliometric Analysis of Spinopelvic Alignment in Total Hip Arthroplasty

**DOI:** 10.5435/JAAOSGlobal-D-22-00182

**Published:** 2023-06-16

**Authors:** Samantha Harrer, Justin A. Magnuson, Gregory R. Toci, Andrew M. Star, Arjun Saxena

**Affiliations:** From the Rothman Orthopaedic Institute at Thomas Jefferson University, (Harrer, Dr. Magnuson, Toci, Dr. Star, Dr. Saxena), Philadelphia, PA (Harrer, Dr. Magnuson, Toci, Dr. Star, and Dr. Saxena) and the Sidney Kimmel College of Medicine at Thomas Jefferson University, Philadelphia, PA (Harrer).

## Abstract

Postoperative dislocation of unclear etiology remains a concern after total hip arthroplasty (THA). Interest is growing in the importance of spinopelvic alignment on stability in THA. The purpose of this study was to analyze publication trends, areas of interest, and future research direction of spinopelvic alignment in THA.

**Methods:**

Articles on the topic of spinopelvic alignment in THA published between 1990 and 2022 were obtained through Web of Science Core Collection of Clarivate Analytics (WSCCA). Results were screened by title, abstract, and full text. The inclusion criterion was English-language peer-reviewed journal publications on the clinical topic of spinopelvic alignment in THA. Bibliometric software was used to characterize publication trends.

**Results:**

We screened 1,211 articles, yielding 132 meeting the inclusion criterion. From 1990 to 2022, published articles have steadily increased, peaking in 2021. Countries that have been the most productive in contributions to research are those in which THA is the most prevalent. Our analysis of keyword frequency showed increasing interest in “pelvic tilt,” “anteversion,” and “acetabular component” position.

**Conclusion:**

Our study identified that increasing attention is being given to spinopelvic mobility and PT in the setting of THA. The United States and France produced the most studies related to spinopelvic alignment.

Despite advances in surgical technique, implant design, and technology that have lowered the dislocation rate in total hip arthroplasty (THA) patients, dislocation and postoperative instability, often of unclear etiology, remain a concern. In 2019, the American Joint Replacement Registry reported instability in association with 17% of hip revision procedures, and in 2020, the Australian Registry listed dislocation as the most frequent reason for hip revision surgery (22%). Current research highlights the inter-relationship of the pelvic and lumbar spine as a possible explanation in prosthetic dislocations of unknown etiology.^1^ Analysis of spinopelvic alignment is particularly relevant as the prevalence of concomitant degenerative hip and spine disease continues to increase.^2^ Questions remain regarding the contribution of spinopelvic alignment to the ideal acetabular implant position and stability. Improved understanding of the complex and dynamic relationship between the spine, pelvis, and hip joint has dispelled the previous theory that the acetabular implant position is static and constant. A growing interest in this relationship has demonstrated the importance of accurate assessment of a patient's spinopelvic parameters in preoperative planning for THA, and this has created a growing body of literature.^3^

Bibliometric analysis is a statistical evaluation of published scientific articles, books, and book chapters. It particularly evaluates the contribution of a research field, including countries/regions, institutions, journals, and researchers to reveal research hot spots and predict future research trends. Critical evaluation of the existing literature on spinopelvic alignment in patients undergoing THA has been limited by disparate terminology and conflicting definitions that confuse the topic of interest.^3^ To date, no bibliometric analysis of spinopelvic alignment in the setting of THA exists in the literature and sparse data are available on trends and future research directions of spinopelvic alignment in patients undergoing THA. Isolated bibliometric analyses of spinal deformities exist, and several peripherally mention spinopelvic alignment as a clinical parameter studied in patients with spinal deformities. Key subjects mentioned in existing spinopelvic analysis include “surgery,” “scoliosis,” and “complications.”^4,5^ However, because growing literature proposes spinopelvic alignment as a critical parameter in THA, especially regarding complications and revision surgery, there is a need to examine the current literature available specifically relating to spinopelvic alignment in THA. The purpose of this study was to analyze publication trends in the existing literature, geographic locations of research productivity, and future research direction of spinopelvic alignment in the setting of THA.

## Methods

### Sources of Data and Search Strategy

The Web of Science Core Collection of Clarivate Analytics was used to conduct our bibliometric analysis. The inclusion criterion was English-language peer-reviewed journal publications on the clinical topic of spinopelvic alignment in THA. Search results were excluded if the topic was not related to spinopelvic alignment or THA.

### Data Collection

Data collection was done using the Web of Science Core Collection of Clarivate Analytics as our main database by two researchers. We searched the database using any form related to spinopelvic alignment and THA using the following search terms: (((ALL=(spinopelvic)) OR ALL=(spino-pelvic)) OR ALL=(hip AND spine))) AND ALL= (arthroplasty).

A total of 1,211 results published between 1990 and 2022 were returned using the search parameters and screened by two authors. Of these, 1,018 were removed by title, 59 by abstract, and two by full text. A final of 132 articles were then included in our bibliometric analysis.

### Bibliometric Analysis

The retrieved data were evaluated by converting screened references into text format and importing into analysis tools, including CiteSpace 5.7.R1, 64-bit (Drexel University), VOSviewer 1.6.15 (Leiden University), and the Online Analysis Platform of Literature Metrology (http://bibliometric.com/). The bibliometric method was used to identify study author characteristics, journals of publication, institutions, countries, keywords, and cocited (two references cited by the same article) articles' networks and keywords.

Cocited reference analysis was also conducted on collected data. Two references cited by the same article are considered cocitations. Frequent cocitation of references suggests a relationship between articles and helps identify research foci of a topic using the bibliometric method.^6^ CiteSpace was used to create visualizations of cocitation networks across included articles for this study.

## Results

### Distribution of Articles by Publication Years

**Figure 1:**
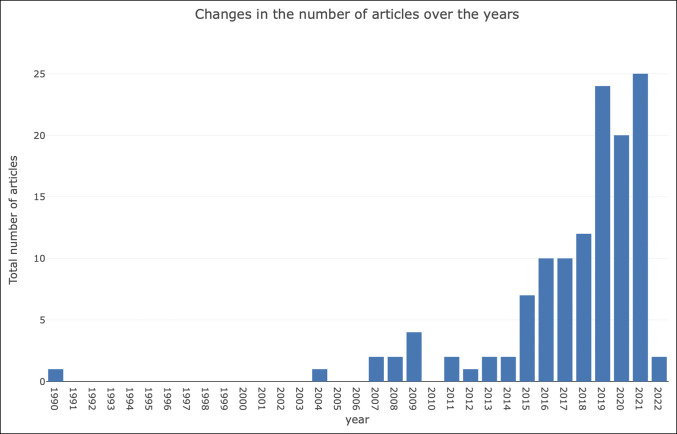
Graph showing trends in the number of publications on spinopelvic alignment in total hip arthroplasty from 1990 to 2022.

From 1990 to 2022, 132 articles were published on spinopelvic alignment in THA (Figure 1). Publication markedly increased beginning in 2015 with an overall upward trend up to 2021. Publication dramatically rose in 2019 with a slight decrease in 2020 and then a peak in 2021. Over 50% of the total publications were produced between 2019 and 2021. Early publication in 2022 thus far suggests steady continued activity in spinopelvic alignment within the context of THA publications.

### Journal Analysis

The top 10 most active journals in spinopelvic alignment in THA are summarized in Table 1. The Journal of Arthroplasty markedly led in publications, with 47 of the 132 articles analyzed in this study. The next most active journal, the Bone and Joint Journal, published 13 of the 132 articles analyzed. The publishers of these journals are predominantly located in the United States and the United Kingdom, followed by Germany and France.

**Table 1:** Top 10 Most Active Journals That Published Articles on Spinopelvic Alignment in Total Hip Arthroplasty

Journal Name	Total No. of Articles	Total No. of References	Average No. of Citation
Journal of Arthroplasty	47	277	5.89
Bone and Joint Journal	13	146	11.23
European Spine Journal	7	31	4.43
International Orthopaedics	6	22	3.67
Clinical Orthopaedics and Related Research	5	50	10
Journal of Bone and Joint Surgery (American Volume)	4	104	26
Orthopaedics and Traumatology: Surgery and Research	4	31	7.75
HIP International	4	21	5.5
Orthopaedic Surgery	3	7	2.3
Revue de Chirurgie Orthopedique et Reparatrice de l’Appareil Moteur	3	2	0.67

In the total number of references, the Journal of Arthroplasty led with 277 and was followed by the Bone and Joint Journal with 146. Clinical Orthopaedics and Related Research led the average number of citations with 110, followed by the Journal of Bone and Joint Surgery (American Volume) with 26. Most of the articles were clinical studies, including case series (57%), cohorts (13%), and case controls (8%). Review articles represented an additional 21% while one randomized controlled trial was identified.

### Contributions of Authors

The top 10 most productive authors of spinopelvic alignment in THA publications are listed in Table 2. Among these, Vigdorchik from the Hospital for Special Surgery (New York, NY) ranked first with 15 publications, followed closely by Buckland from New York University Langone Health (New York, NY) and the Melbourne Orthopaedic Group (Melbourne, Australia) with 14 publications. Schwarzkopf from New York University Langone Health (New York, NY) had the highest average number of citations with 220, followed by Vigdorchik with 180.

**Table 2:** The Top 10 Most Productive Authors of Spinopelvic Alignment in Total Hip Arthroplasty Research

Author's Name	Total No. of Articles	Total No. of References	Average No. of Citations	No. of Times Work Cited	No. of Corresponding Authors
Vigdorchik, JM	15	120	18.00	9	7
Buckland, AJ	14	175	12.50	93	7
Dorr, LD	8	130	16.25	0	5
Schwarzkopf, R	7	154	220.00	0	2
Grammatopoulos, G	7	25	3.50	10	5
Mayman, DJ	6	71	11.83	0	0
Lazennec, JY	6	41	6.83	27	2
Abdel, MP	6	26	4.30	1	4
Beaule, PE	6	24	4.00	0	1
Innmann, MM	6	15	2.50	15	0

### Country/Region and Institution Analyses

**Figure 2:**
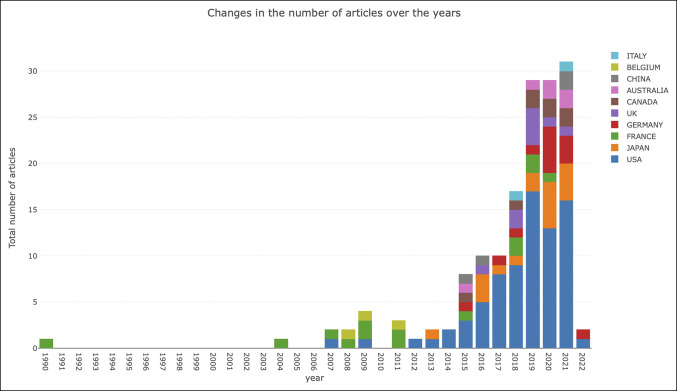
Graph showing top 10 countries conducting research on spinopelvic alignment in total hip arthroplasty from 1990 to 2022.

Publication numbers per country by year on spinopelvic alignment in THA are presented in Figure 2 for the top 10 most active countries. In 1990, France initiated research on spinopelvic alignment in THA and led in publications until 2011, with an exception in 2007 when the United States and France published an approximately equivalent number of articles. In 2012, the United States then published the most research on spinopelvic alignment in THA, and in 2013, the United States and Japan had roughly equivalent publication activity. Then, between 2014 and 2021, the United States was the largest contributor to publications on spinopelvic alignment comprising nearly 50% per year of published articles. Thus far in 2022, the United States and Germany are leading in active publications.

Regarding activity of the top 10 most productive research institutions, the Hospital for Special Surgery ranked first with 26 articles, followed by New York University with 14 articles and the Mayo Clinic with nine articles. The top three contributing research institutions are located in the United States, with two Japanese research institutions within the top seven most productive institutions. In addition to the United States and Japan, the Imperial College of London and the Melbourne Orthopaedic Group comprise the most active research institutions (Table 3).

**Table 3:** The Top 10 Institutions Contributing to Publications in Spinopelvic Alignment in Total Hip Arthroplasty

Institution Name	Total No. of Articles	Total No. of References	Average No. of Citations	No. of Times Work Cited
Hospital for Special Surgery	26	180	6.90	100
New York University	14	221	15.70	116
Mayo Clinic	9	58	6.40	18
Yokohama City University	8	45	5.63	18
University of Louisville	6	56	9.30	26
SUNY Downstate Medical Center	6	94	15.67	36
Kyoto University	5	0	0.00	0
New York University Langone Orthopedic Hospital	5	6	1.20	5
Imperial College of London	5	13	2.60	7
Melbourne Orthopaedic Group	5	19	3.80	0

The research network map among institutions showed a centralized density of contributors, indicating collaboration between institutions. Collaboration between the United States and Australia occurred most frequently, followed by collaborations between the United States and Japan, France and Belgium, and France and the United Kingdom.

### Keyword Occurrence Cluster Analysis of Research Hot Spots

**Figure 3:**
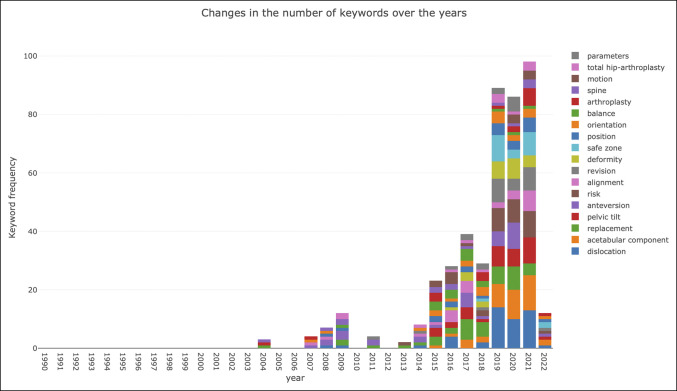
Graph showing keyword frequency changes by year in spinopelvic alignment in total hip arthroplasty research.

**Figure 4:**
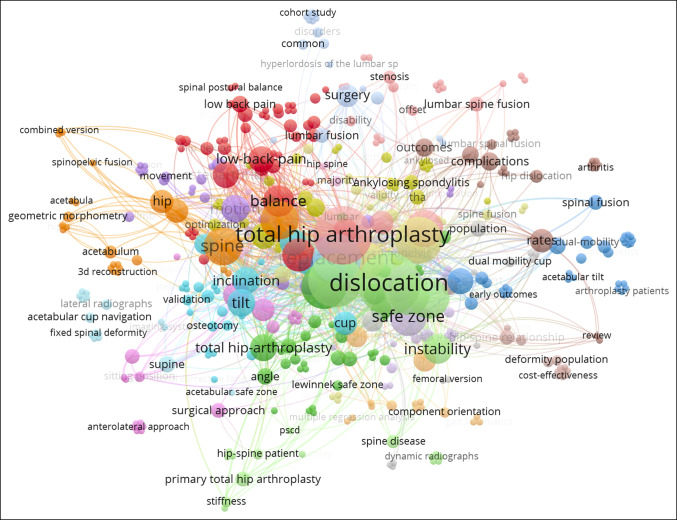
Illustration showing keyword cluster analysis of spinopelvic alignment in total hip arthroplasty research.

Changes in publication keyword occurrence by year are illustrated in Figure 3. A total of 17 keywords were identified in our analysis of publications on spinopelvic alignment in THA: “parameters,” “total hip arthroplasty,” “motion,” “spine,” “arthroplasty,” “balance,” “orientation,” “position,” “safe zone,” “deformity,” “revision,” “alignment,” “risk,” “anteversion,” “pelvic tilt,” “replacement,” “acetabular component,” and “dislocation.” Before 2014, distribution of keyword frequency was relatively even, with “anteversion” being the most frequently used keyword for several years. Then in 2015, “balance,” “spine,” and “replacement” became increasingly used. Most recently, “dislocation” and “acetabular component” have the top frequency of keywords from 2019 to 2021. Thus far in 2022, a relatively even split of multiple keywords has been observed. Visualization of keyword cluster analysis is presented in Figure 4. Data analysis demonstrates 17 clusters among keywords.

### Top Cocited Articles and Cocited Reference Cluster Analysis

**Figure 5:**
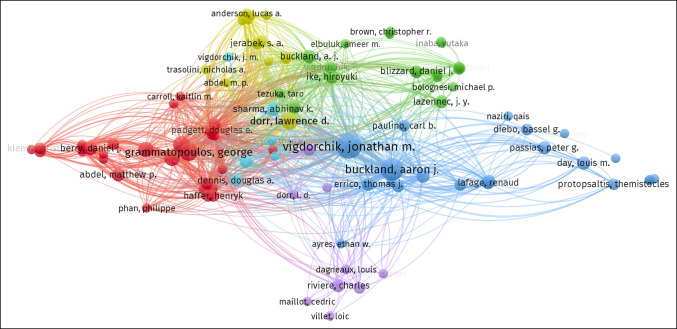
Illustration showing the citation network of authors of spinopelvic alignment in total hip arthroplasty research.

**Figure 6:**
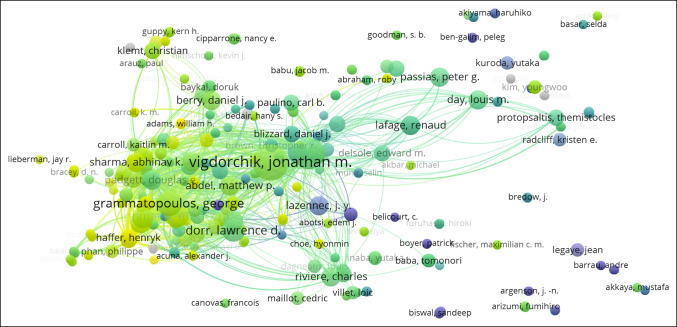
Illustration showing cocited reference cluster analysis of spinopelvic alignment in total hip arthroplasty (THA) research.

Analysis of cocitation between authors was conducted with results illustrated in Figure 5. This visualized citation network between authors consists of a single node for a cited author. Each link between the nodes demonstrates citation between authors with an increased frequency of citation demonstrated by an increased density of the line. Furthermore, the diameter of each node is proportional to the total cocitation count of the associated author. Visualization of cocited reference cluster analysis articles is illustrated in Figure 6. Our data demonstrate 9 clusters and 20,507 links.

**Figure 7:**
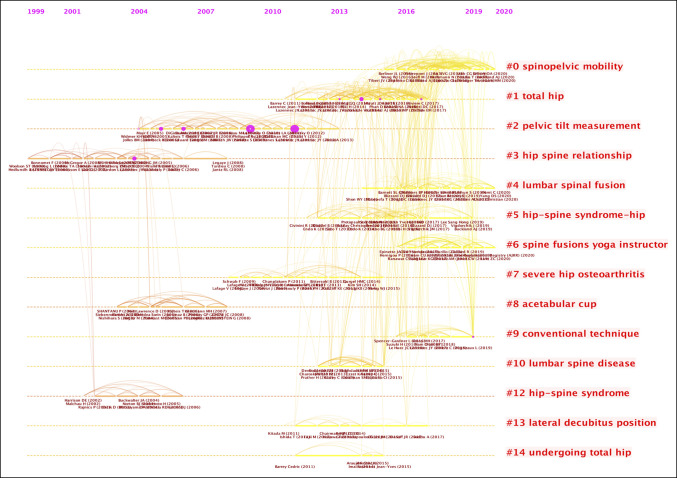
Diagram showing the time-line view of cocitation clusters with cluster labels of spinopelvic alignment in total hip arthroplasty research.

Additional cluster analysis conducted on the cocited references demonstrates 14 clusters, including spinopelvic mobility, total hip, hip-spine relationship, pelvic tilt (PT) measurements, lumbar fusion, and hip-spine syndrome. Figure 7 demonstrates the research clusters from a time-line perspective, helping to identify emerging research foci in spinopelvic alignment in THA.

## Discussion

This study used bibliometric analysis to summarize publications between 1990 and 2022 that reported the relationship of spinopelvic alignment on THA outcomes. During these years, the number of publications on spinopelvic alignment in THA steadily increased, reaching a peak of 25 publications per year in 2021. For 2022, publication activity has remained consistent.

Spinopelvic mobility and the relationship with hip dislocation were first suggested in 2015.^6^ As a patient moves from standing to sitting, the sacrum tilts posteriorly and lumbar lordosis decreases. This causes the acetabulum to flex, which reduces the likelihood of impingement of the rim of the acetabulum on the femur as the hip flexes. Spinal stiffness decreases the ability of the pelvis to adjust as a patient changes position, increasing the likelihood of impingement.^6,7^ Furthermore, classifications of spinopelvic mobility have been developed based on flexibility and sagittal balance and include the following: (1) flexible and balanced, (2) flexible and unbalanced, (3) rigid and balanced, and (4) rigid and unbalanced.^6,8^ Additional classifications of the spinopelvic relationship have been proposed. For example, Kanawade et al. categorized patients into three groups based on radiographic measures of pelvic motion from sitting to standing.^9^ These groups included normal pelvic motion, stiff pelvis, and a hypermobile pelvis.^9^ Stefl et al. developed an additional classification based on the findings of Kanawade et al. with expansion of the stiff pelvis group into anterior tilt, posterior tilt, kyphotic, or fused.^10^ Finally, the Hip-Spine Classification includes four categories based on spinal stiffness and spinal deformity with classification as follows: 1A: normal spinal alignment and mobility; 1B: normal spinal alignment and stiff spine (defined as <10° ΔSS); 2A: flatback spinal deformity (pelvic incidence–lumbar lordosis [PI-LL] ≥10°) and normal mobility; and 2B: flatback deformity and stiff spine.

Normal spinal mobility is defined as >10° ΔSS (change in sacral slope from standing to sitting) while normal alignment is defined as PI-LL<10° (difference in the pelvic incidence and lumbar lordosis).^11^

Based on the category of spinopelvic mobility and the spinopelvic relationship, variations in cup implantation and implant selection have been proposed.^8,9,10,11^ Outcome studies examining the efficacy of implant changes based on spinopelvic relationship categorization have been relatively few. Data collected by Vigdorchik et al. suggested poor outcomes in using dual-mobility implants for the 2B classification as proposed by the Hip-Spine Classification.^12^

Countries that have been most productive in contributions to research on spinopelvic alignment are those in which THA is increasingly prevalent, including Germany, Austria, Switzerland, France, and the United States.^12,13^ However, the analysis did not show a consistent relationship between THA volume and collaboration between countries. Collaboration was demonstrated between the United States and France as well as France and Belgium, but not Switzerland and other countries with high rates of THA. Israel, a country with lower total joint arthroplasty rates, did show collaboration with the United States.^12^ A lack of a predictable association between collaboration and joint volumes suggests that collaboration is influenced by other factors that were not elucidated by this bibliometric analysis. It should not be surprising that the most active authors conducting research on spinopelvic alignment in THA are from countries with a large THA volume and high projected growth.^13,14^ This is consistent with intense interest in the contribution of spinopelvic alignment to successful THA outcomes for these authors.

As previously described, instability and dislocation are leading causes for revision surgery after THA. Revision THA poses notable stress to patients, with decreased quality of life documented as well as increased stress to surgeons and the healthcare system for markedly increased costs.^15,16^ Furthermore, dislocations of unclear etiology after THA comprise a notable portion of those requiring revision surgery, ranging between 7 to 18%.^17^ Our analysis of keyword frequency and changes in the existing literature over the past 3 decades has demonstrated the frequent use of the terms “dislocation,” “pelvic tilt,” “anteversion,” “acetabular component,” “sacral slope,” “safe zone,” “impingement,” and “hip-spine syndrome.” These findings suggest that increased attention is necessary in patients with concurrent spine pathology to ensure optimal implant placement. Surgeons should be aware of potential increased risk of dislocation in these patients. Abdel et al^18^ reported that conventional parameters for acetabular implant positioning were not valid and that there were other factors that were necessary to predict postoperative dislocation. This bibliometric analysis would suggest that parameters such as PT and acetabular position play a notable role in our future understanding of optimal performance of total hip arthoplasty. PT directly affects the orientation of the acetabular cup and has been studied preoperatively and postoperatively in patients undergoing THA. Alterations in spinopelvic mobility spinal sagittal alignment brought about by lumbar disk disease and lumbar fusion are risk factors of postoperative dislocation and revision.^7,19,20^ Furthermore, research has demonstrated that PT and acetabular implant anteversion angle are directly related and acetabular anteversion malpositioning can lead to increased prosthesis wear, which may cause late hip prosthesis dislocation.^21,22^ The fine-tuning of the most frequently used keywords in the published literature highlights elucidation of the problem at hand and precise efforts toward defining an understanding.

Spinopelvic alignment is of varying importance in different patient populations; however, our investigation looked into the topic from a general perspective without specifying any subgroups, which may represent a limitation to our analysis. Because of the previously described findings, increasing attention is being given to spinopelvic mobility and PT in the setting of THA. Our analysis suggests continued scholarly inquiry into these topics with particular focus on the influence of spinopelvic mobility and PT on THA outcomes, with the goal of further reducing postoperative dislocation and need for revision.

## Conclusion

Our bibliometric analysis allowed for novel exploration and identification of the current research trends, the contribution of research to this field, and the distribution of publications exploring spinopelvic alignment in the setting of THA to predict future research trends and identify research hot spots.
